# Expansion Kinetics of Flexible Polymers upon Release from a Disk-Shaped Confinement

**DOI:** 10.1021/acsomega.3c08378

**Published:** 2024-03-08

**Authors:** Pai-Yi Hsiao

**Affiliations:** † Department of Engineering and System Science, National Tsing Hua University, Hsinchu, Taiwan 30013, R.O.C; ‡ Institute of Nuclear Engineering and Science, National Tsing Hua University, Hsinchu, Taiwan 30013, R.O.C

## Abstract

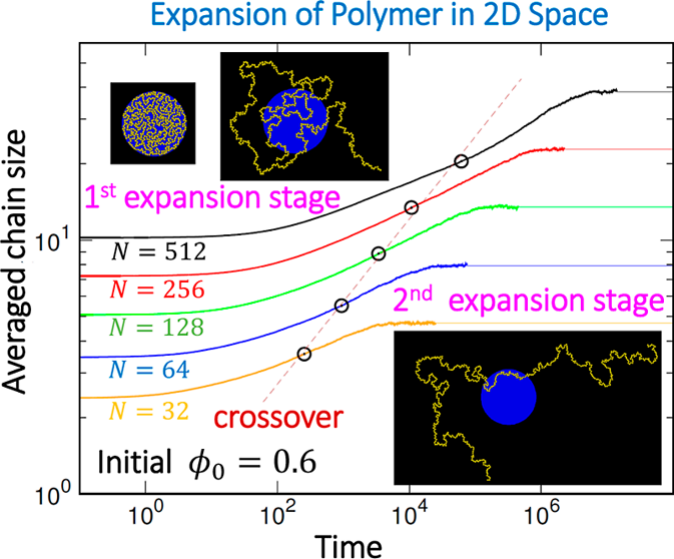

A general theory is developed to explain the expansion kinetics of a polymer released from a confining cavity in a *d*-dimensional space. At beginning, the decompressed chain undergoes an explosive expansion while keeping the structure resembling a sphere. As the process continues, the chain transitions to a coil conformation, and the expansion significantly slows down. The kinetics are derived by applying Onsager’s variational principle. Computer simulations are then conducted in a quasi-two-dimensional space to verify the theory. The average expansion of the chain size exhibits a distinctive sigmoidal variation on a logarithmic scale, characterized by two times and associated exponents that represent the fast and the slow dynamics, respectively. Through an analysis of the kinetic state diagrams, two important universal behaviors are discovered in the two expansion stages. The intersection of the expansion speed curves allows us to define the crossover point between the stages and study its properties. The scaling relations of the characteristic times and exponents are thoroughly investigated under different confining conditions, with the results strongly supporting the theory. Additional calculations conducted in a three-dimensional (3D) space demonstrate the robustness of the proposed theory in describing the kinetics of polymer expansion in both 2D and 3D spaces.

## Introduction

The release of biomacromolecules from a confined state is a fundamental process in microbiology. For example, viral genomes can be released from uncoated capsids after viruses enter host cells to cause infection.^1^ In various applications, genetic materials or drugs can be encapsulated in small particles and later released at targeted locations for therapeutic purposes.^2,3^ A comprehensive understanding of the release kinetics is crucial for successfully designing therapy protocols. Despite its significance, our current knowledge of this phenomenon remains limited. One relevant area of research is the study of the globule-to-coil transition, which examines the state transition of a polymer chain due to changes in solvent quality.^4^ While the former situation involves confining chains through external means such as cavity walls, the latter is caused by internal attractions that lead to chain collapse due to poor solvent conditions. As a result, the globular structures before release differ in these two situations, potentially resulting in different release kinetics.

Recent experiments have shown that evolution of chain size in a globule-to-coil transition cannot be adequately described by a simple recovery function.^5,6^ The expansion process is characterized by a phenomenological function that involves a stretched exponential exp(−(*t*/τ)^β^) with β < 1. A comprehensive explanation for the kinetics is still lacking. Theoretical studies related to macromolecule collapse transitions can be traced back to the works of De Gennes et al.^7^ and Grosberg et al.^8^ in the 1980s. Pitard and Orland later investigated the swelling behavior and proposed two kinetics: a power-law-like growth first, followed by an exponential recovery.^9^ Sakaue and Yoshinaga balanced the change in free energy with the dissipated energy and derived the kinetics under the assumption of spherical expansion.^10^ They predicted different power-law growths for chain size. Lee et al. discussed the role of entanglements in a chain globule and also found power-law growth for the chain expansion.^11,12^ Doyle and co-workers experimentally showed that the evolution of chain size should exhibit exponential recovery.^13^ The decompression of single DNA molecules in a nanochannel has been investigated, revealing a modified recovery powered by an exponent of 1/3.^14^ However, the results of these studies are not always consistent, and a compelling theory is currently lacking to resolve the controversy. One of the main difficulties stems from the strong stochastic nature associated with an expansion process. Typically, tens of measurements for single-chain expansion curves are not sufficient to extract the kinetics with high precision.

Recently, we have developed a two-stage model to explain the expansion of polymers released from a spherical cavity in a three-dimensional (3D) space.^15^ In the model, the chains undergo a rapid spherical expansion in the first stage, followed by a dominant coil-like expansion in the second stage. Importantly, the growth in the latter stage exhibits a hastened exponential recovery, powered by an exponent of 1/5. The work presented here aims to broaden our knowledge from a 3D space to a two-dimensional (2D) one. However, since the expansion of a compressed chain upon release is a nonequilibrium process, extending the work to other spatial dimensions is not so obvious and requires careful verification of its applicability. Moreover, the new analysis presented here reveals two important universal behaviors in the kinetic state diagrams. These diagrams will provide valuable insights into the underlying behaviors of the expansion process.

## Theory and Simulation Setting

A generalized theory is developed in the *d*-dimensional space. The chain is assumed to be flexible because viral genomes or biopolymers in applications can consist of single-stranded RNA or DNA molecules, which are considerably flexible. The modeled chain is composed of *N* beads, each of which has a diameter of σ, connected by bonds of length σ as well. The diameter of the confining cavity is *D*, which gives a volume fraction of  for the chain. The expansion is started by removing the confining wall of the cavity. Initially, the chain expands with a conformation resembling a sphere for *d* = 3 or a disk for *d* = 2. It is called the spherical-expansion stage. The chain adopts a coil-like structure later, and the expansion continues until reaching the final chain size in the bulk solution. This is the coil-expansion stage.

The kinetic equation governing the expansion can be derived by applying Onsager’s variational principle.^16^ By balancing the change in free energy *F* of the chain with the energy dissipation through friction with the solvent, the equation can be written as
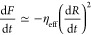
1where *R* and  are two state variables representing the chain size and the expansion speed at time *t*, respectively, and η_eff_ denotes the effective friction coefficient associated with the expansion speed.

In the first stage, the free energy *F* can be estimated by using the blob theory, which assumes the maintenance of a spherical conformation of the chain.^17,18^ It is expressed as , where ν_b_ is an exponent that relates the size ξ_b_ of a blob to the number *g*_b_ of monomers in the blob through . By solving the kinetic equation  with the given initial chain size *R*_0_, the evolution of chain size can be determined as
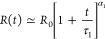
2Here,  is the exponent, and  is the characteristic time with *A*_1_ being a prefactor. The effective friction η_eff_ has been assumed to scale as  in the derivation, where η_0_ represents the friction coefficient for a single monomer. The exponent χ_1_ takes into account the additional scaling dependence on the chain length because the rate of energy dissipation is described by using the square of the state variable  in eq 1, rather than the sum of the squares of the speeds of individual monomers.^19^ The details of the derivation can be found in the Supporting Information, Section S1.

In the second stage, the flexible chain expands by adopting a coil conformation. The Flory free energy  is used in this situation.^20^ The corresponding kinetic equation is now a Bernoulli differential equation: . The time variation of the chain size is solved
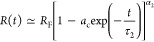
3where *R*_F_ denotes the final chain size. It is an exponential recovery function powered by an exponent , which accelerates the progress of expansion. The characteristic time τ_2_ is given by , where χ_2_ is an exponent that accounts for the additional dependence on the chain length through  in the second stage. The constant *a*_c_ is proposed to be set as 1 based on the simulations presented later and our previous study in 3D space.^15^ This choice is applicable for the situation of a long chain released from strong confinement and turns out to be a good approximation for general situations. It will be explained later.

The predicted exponents and characteristic times for the chain expansion in 2D and 3D spaces are summarized in Table 1.

**Table 1:** Predicted Exponents and Scaling Formulas for the Characteristic Times of Chain Expansion in *d*-Dimensional Space^a^

	formula		*d* = 2		*d* = 3	
	strong conf	weak conf	strong conf	weak conf	strong conf	weak conf
ν_b_	1/2	3/(*d* + 2)	1/2	3/4	1/2	3/5
α_1_	(dν_b_ – 1)/(2(dν_b_ – 1) + *d*)		0	1/6	1/8	4/23
τ_1_	N^(2/*d*) + χ_1_^ϕ_0_^–((2/*d*) + (1/(dν_b_ – 1)))^		N^1 + χ_1_^ϕ_0_^–∞^	N^1 + χ_1_^ϕ_0_^–3^	N^(2/3) + χ_1_^ϕ_0_^–8/3^	N^(2/3) + χ_1_^ϕ_0_^–23/12^
α_2_	1/(*d* + 2)		1/4		1/5	
τ_2_	*N*^2 + χ_2_^		*N*^2 + χ_2_^		*N*^2 + χ_2_^	

a Different ν_b_ values are used in the calculations for strong and weak confinement scenarios.

The exponent ν_b_, depicting the scaling of the blob size in a cavity, depends on the degree of confinement. In a strong confinement, where the chain is highly compressed and resembles a melt, the value of ν_b_ is about 0.5. However, under a weak confinement, ν_b_ takes on the value of the Flory exponent ν, which is  in a *d*-dimensional space. Consequently, α_1_ and τ_1_ are determined by the two situations, as given in the table.

To verify the theory, molecular dynamics simulations are performed in a quasi-2D space created within a slit region of height *H*. Initially, a bead–spring chain is confined and equilibrated in a disk-shaped cavity with a diameter of *D*. The apparent volume fraction of chain is equal to , which corresponds to a 2D volume fraction . The excluded volume interaction between beads is modeled by Weeks–Chandler–Anderson potential with a strength of ε = 1.2*k*_B_*T*,^21^ where *k*_B_ denotes the Boltzmann constant. The bonding is modeled by a harmonic spring, which connects through the bead centers. The spring constant and the equilibrium bond length are set as *k* = 6000*k*_B_*T*/σ^2^ and *b*_0_ = σ, respectively. In the simulations, the temperature is controlled by using a Langevin thermostat. It is an isothermal technique that implicitly simulates solvents by incorporating random forces based on the fluctuation–dissipation theorem.^22^ The top and bottom walls of the slit, as well as the side wall of the cavity, are assumed to be reflective. Once the side wall is removed, the chain starts to expand and eventually reaches its natural size within the slit region. The dynamics of the system are simulated using LAMMPS.^23^ The integration time step is . The chain length is varied from *N* = 16–512. For each selected chain length, four different ϕ_0_ values are studied, specifically ϕ_0_ = 0.6, 0.3, 0.15, and 0.075. The height of the slit is set to be *H* = 1.8σ.

Details of the simulation can be found in Section S2 of the Supporting Information, Figures S1 and S2. Snapshots illustrating the release behaviors are given in Figure S3 for a chain with *N* = 512 released from ϕ_0_ = 0.6. The top-view images show that the chain initially expands with a disk-like structure and gradually transitions into a coil conformation over time. Figure S4 presents the probability density distribution of the bond length in the simulations. It shows that the passage of a chain portion from a cleavage space formed by two connected monomers on the chain within the slit region is highly unlikely to occur under the simulation conditions. The author did not detect any such event in any of the runs.

In the following text, the results will be reported by using the simulation units: *m*, *k*_B_*T*, and σ. The error bars of the data are not shown explicitly because they are smaller than the size of the plotted symbols on the figures.

## Results and Discussion

**Figure 1:**
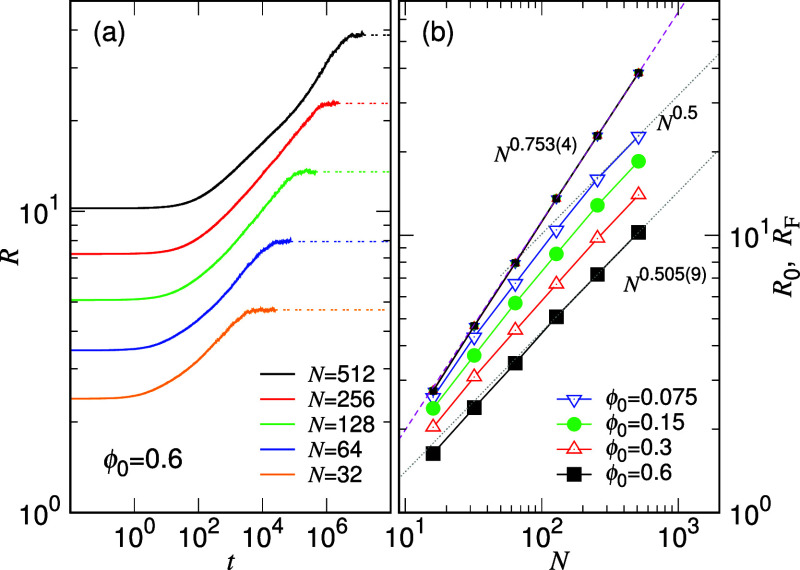
(a) Time evolution of *R* for different chain lengths *N* (indicated in the legend), released from the confining condition ϕ_0_ = 0.6. (b) Initial chain size *R*_0_ (with large data symbols) and final chain size *R*_F_ (with small symbols) plotted against *N*. The value of ϕ_0_ can be read from the legend.

The mean radius of gyration is utilized to characterize the chain size, calculated by , where **r**_*i*_ is the position of the *i*th monomer and **r**_cm_ denotes the center of mass of the chain. The time evolution *R*(*t*) is obtained by averaging over 1000 independent runs. The results are presented in Figure 1a for different *N* values where the confining condition is ϕ_0_ = 0.6.

In the log–log plot, *R*(*t*) is displayed as a sigmoid function, transitioning from the initial size *R*_0_ within the disk confinement to the final size *R*_F_ in the slit space. Notably, the variations appear to be similar to each other, suggesting the existence of some underlying scaling properties over the chain length. The same plot on linear scales can be found in Figure S5. The chain expansion occurs rapidly at the beginning, while the recovery back to its natural size is considerably slower and spans over a wide time range, depending on the chain length. Therefore, studying the evolution on logarithmic scales is more suitable for capturing and analyzing the kinetics. Figure S6 presents the average curves obtained from 10, 100, and 1000 runs, showing the necessity of a large number of samples for the accurate analysis of the evolution behavior.

To verify the simulations, the scaling property of *R*_0_ versus that of *N* is studied. As shown in Figure 1b with the large data symbols, *R*_0_ exhibits a scaling behavior of *N*^0.505(9)^ for ϕ_0_ = 0.6, as expected for a chain confined in a disk region. Decreasing ϕ_0_ implies a looser confinement, resulting in an increase in the chain size. Consequently, the scaling exponent deviates from 0.5, especially in the region of small *N* where *R*_0_ approaches the final chain size *R*_F_, as indicated by the small symbols in the figure. Because the final sizes are the same at a given *N*, the small symbols overlap with each other, regardless of the value of ϕ_0_. The scaling for *R*_F_ is found as *N*^0.753(4)^, consistent with the predicted exponent 0.75 from Flory’s theory for a 2D space.

The scaling behaviors of *R*(*t*) are investigated by analyzing the two quantities:  and . The first quantity measures the expansion ratio relative to the initial size and has a starting value of 1. It exhibits a similar bending-up behavior for different *N*. To demonstrate the similarity, the  curve for *N* = 2^*g*^ is horizontally shifted by multiplying time by *a* factor of ω_1_^9-*g*^. A good choice of the scaling parameter ω_1_ will collapse the shifted curves onto the targeted one for *N* = 2^9^ = 512 in making the plot against *t*_ω_1__ = *t* × ω_1_^9-*g*^. The choice is determined by searching for the minimum mean width ⟨*W*_1_⟩ of collapse of the set of the shifted curves in the early time region, as explained in Figures S7 and S8 of Supporting Information.

**Figure 2:**
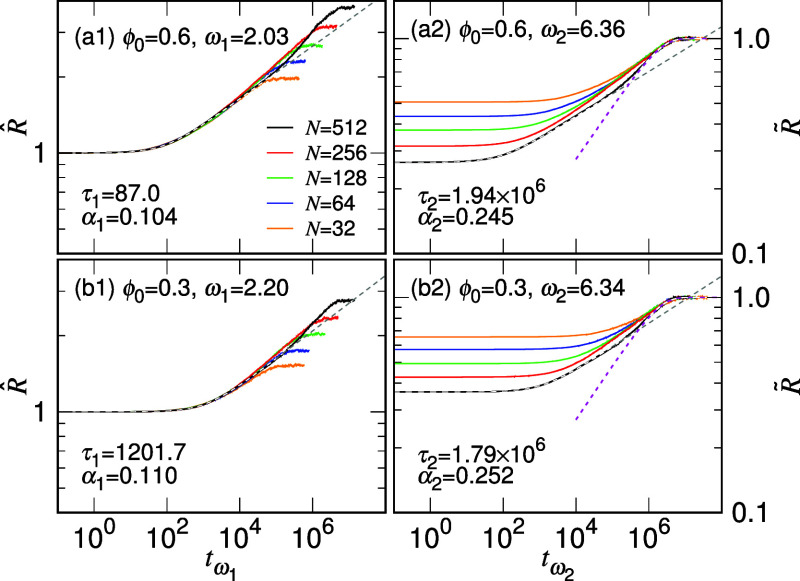
vs *t*_ω_1__ = *t* × ω_1_^9-*g*^ and  vs *t*_ω_2__ = *t* × ω_2_^9-*g*^ for (a1, a2) ϕ_0_ = 0.6 and (b1, b2) ϕ_0_ = 0.3. The value of *N* can be read in the legend of panel (a1). The optimal ω_1_ and ω_2_ values for the best collapse and the fit parameters α_1_, τ_1_, α_2_, and τ_2_ are reported in the corresponding panels. The fitting curves for the first and the second expansion stages are plotted as gray and magenta dashed lines, respectively.

Illustrations of the best collapses are provided in Figure 2, parts a1 and b1, for ϕ_0_ = 0.6 and 0.3, respectively.

Enlarged plots for a clearer visualization of them can be found in Figure S9. The optimal ω_1_ values for achieving the best collapse are 2.03 and 2.20 for the two cases. Additional plots showing the collapses for ϕ_0_ = 0.15 and 0.075 are given in Figure S10. The collapsed curves are fitted using eq 2. The resulting τ_1_ and α_1_ values are reported in the corresponding panels. The fitting curves are shown in gray dashed lines for comparison.

The second quantity  measures the percentage of the chain size that has completed the expansion. Similarly, the curves can be collapsed by plotting them against the time scale *t*_ω_2__ = *t* × ω_2_^9-*g*^. The ω_2_ values for the best collapse are 6.36 and 6.34, respectively, for the two cases shown in panels (a2) and (b2) of Figure 2. The procedure to find the best collapse is also explained in Figures S7 and S8. Since ω_2_ is much larger than ω_1_, the expansion process exhibits two distinct kinetics. By fitting the collapse using eq 3 with *a*_c_ = 1, the parameters τ_2_ and α_2_ are obtained. The fitting curves, displayed as magenta dashed lines, demonstrate a good fit with the data. The choice of *a*_c_ = 1 is used in the analysis based upon the observations of simulation. The size evolution during the second expansion stage can be well described by a limiting curve, representing a long chain released from a strong confinement, as explained in the Supporting Information for Figure S11.

**Figure 3:**
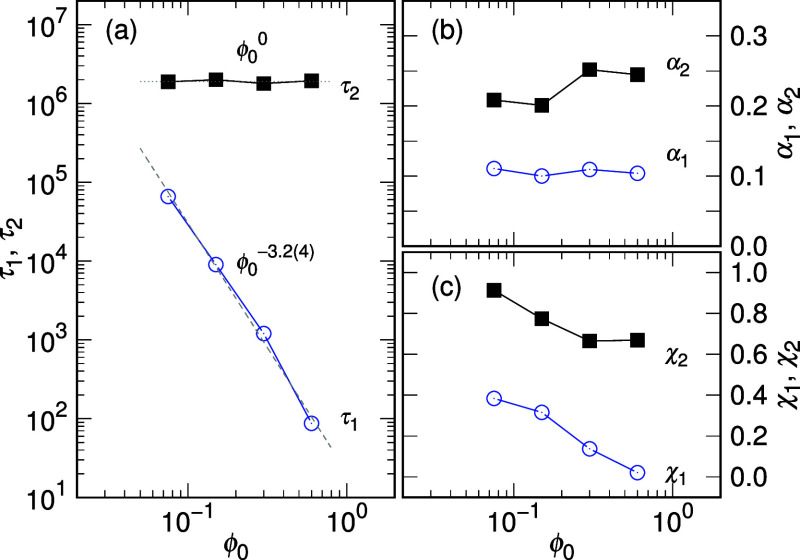
(a) τ_1_ and τ_2_ for *N* = 512, (b) α_1_ and α_2_, and (c) χ_1_ and χ_2_, plotted against ϕ_0_. The characteristic times τ_1_, τ_2_ and the exponents α_1_, α_2_ are parameters defined in eqs 2 and 3. The exponents χ_1_ and χ_2_ are introduced to account for the additional chain length dependence in η_eff_.

The scaling properties for τ_1_ and τ_2_ are studied in Figure 3a for *N* = 512.

It is observed that τ_1_ scales as ϕ_0_^–3.2(4)^, which agrees with the predicted scaling ϕ_0_^–3^ given in Table 1 under the fixed-*N* condition with ν_b_ = 0.75. The characteristic time τ_2_ is found to be not sensitive to ϕ_0_. It is several orders larger than τ_1_, particularly when ϕ_0_ is large. Consequently, the expansion time τ_e_ is primarily determined by τ_2_ and can be defined, for example, as triple of τ_2_, representing the time needed for a 98.7% recovery to the natural chain size.

The exponent α_1_ is about 0.104 and is not sensitive to changes in ϕ_0_, as shown in Figure 3b. α_2_ acquires the predicted 2D value of 0.25 for ϕ_0_ = 0.6 and 0.3 but decreases as ϕ_0_ becomes smaller. This could be attributed to the situation in which *R*_0_ is close to *R*_F_, causing the second stage to mix with the first stage. The exponents χ_1_ = log_2_(ω_1_) – 1 and χ_2_ = log_2_(ω_2_) – 2 describe the additional chain length dependence on the effective friction coefficient. Both exponents increase as ϕ_0_ decreases, as shown in Figure 3c.

In this study, chain size *R* serves as a transition coordinate to describe the progress of an expansion. The kinetics can thus be examined by calculating the time derivative of *R* from the simulations. Our theory predicts
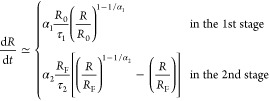
4

**Figure 4:**
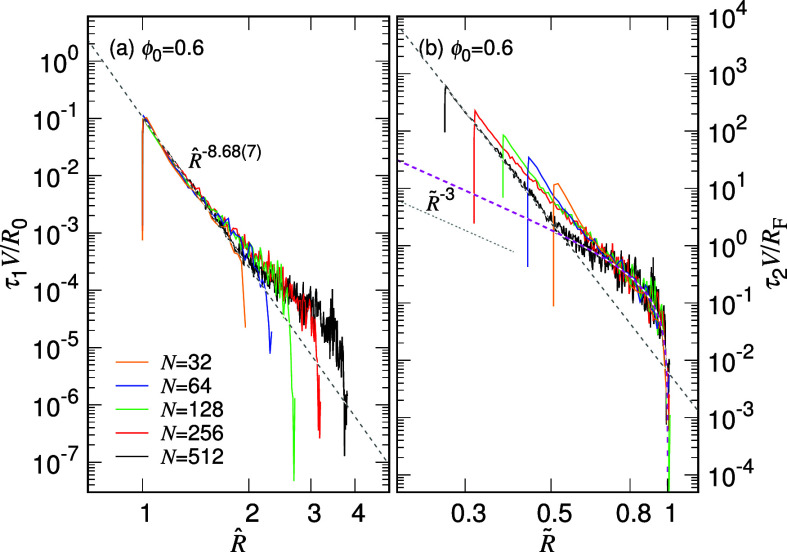
Kinetic state diagrams, (a) τ_1_*V*/*R*_0_ vs  and (b) τ_2_*V*/*R*_F_ vs , for ϕ_0_ = 0.6. The chain length *N* can be read from the legend in panel (a). Note that  and  are equivalent to  and , respectively, because , , , and .

The dimensionless speed , or equivalently τ_1_*V*/*R*_0_, is expected to exhibit a universal power-law behavior in the first stage as , where  denotes the expansion speed and . Figure 4a presents the calculated results for ϕ_0_ = 0.6.

The speed curves do collapse together for different chain lengths in the small  region. The displayed scaling exhibits an exponent of −8.68(7), which corresponds to α_1_ = 0.103(1) according to eq 4. The result is consistent with the findings in Figure 2a1.

The dimensionless speed suitable for the second stage of expansion is , or equivalently τ_2_*V*/*R*_F_, which is expected to display universality in the large  region. As shown in Figure 4b, the collapsed curves can be well described by  with , represented as a magenta dashed line. The magenta curve reveals an asymptotic behavior of  as it extends toward small , and the overall profile resembles a hip curve on the logarithmic scale as  approaches 1. Due to the smaller value of α_1_ compared to α_2_, a transition in the slope can be observed, for example, at  ≈ 0.5 for the case of *N* = 512. These two types of plots shown above will be referred to as the kinetic state diagrams because they depict the kinetic states of chain expansion through the two state variables of chain size and expansion speed.

Additional plots,  vs  and  vs , are presented in Figure S12, allowing for better visualization of the individual kinetic curves. It is observed that the kinetic curves for different *N* join the corresponding magenta dashed curves at different moments. For shorter chains, the crossover occurs later with a larger  value. Since the expansion speed approaches zero as  approaches 1, the slope of the curve decreases significantly. As a result, the transition of slope at the crossover becomes less pronounced for shorter chains, compared to the long chain case of *N* = 512. This phenomenon is termed the finite size effect.

**Figure 5:**
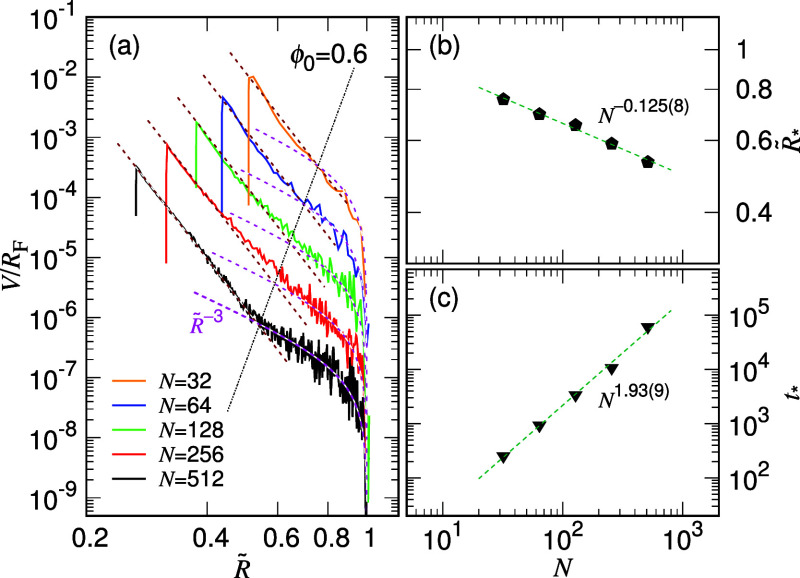
(a) Kinetic state diagram of d/d*t* (or equiv *V*/*R*_F_) vs . The intersect between the two expansion speeds, given in eq 4 by the brown and magenta dashed lines, respectively, allows for the determination of the crossover. The crossover points for different *N* align approximately on a line, plotted as a black dotted line. The confining condition is ϕ_0_ = 0.6. (b) Obtained crossover size  plotted as a function of *N*. (c) t_*_ vs *N* where the crossover time t_*_ is determined by mapping the associated size R_*_ on the *R*(*t*) curves given in Figure S13.

The crossover between the first and the second stages of expansion can be determined by finding the intersection of the two variation behaviors at a specific *N*, given by the set of the brown and magenta dashed lines in Figure 5a of the kinetic state diagram  vs .

It is found that the crossover points align approximately on a line on the log–log scale, as plotted by the black dotted line. On the left of the dotted line is the domain for the first expansion stage, while the right is the one for the second expansion stage.

The obtained crossover size  is plotted in Figure 5b as a function of *N* and exhibits a scaling behavior . Based on eq 4, the equality of the expansion speed at the crossover imposes a relation for  as
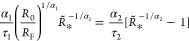
5The “–1” term on the right-hand side of the relation can be omitted if the value of  is well smaller than one. It yields the scaling relation:  with . Given α_1_ = 0.104(2), α_2_ = 0.245(9), χ_1_ = 0.021(7), χ_2_ = 0.669(2), and ν = 0.75 in this study for ϕ_0_ = 0.6, the predicted exponent *x*_R_ has a value of −0.137(14). This result agrees with the scaling observation in Figure 5b, demonstrating the consistency of the theory.

The crossover time t_*_ can be further determined by locating the associated chain size R_*_ on the *R*(*t*) curve, as shown in Figure S13. Figure 5c indicates that t_*_ also follows a power-law behavior, *N*^*x*_*t*_^, with *x*_*t*_ = 1.93(9). Notably, the crossovers on the different *R*(*t*) curves in Figure S13 nearly align on a straight line on the log–log scale. It separates the expansion stages with the first stage on the left and the second stage on the right. The demarcation line is found to exhibit a scaling relation R_*_ ∼ t_*_^0.326(19)^.

The aforementioned procedures are further utilized to reanalyze the simulation data for polymer expansion in a 3D space,^15^ aiming to test the generality of the theory. The collapses of the  and  curves, the scaling behaviors of τ_1_ and τ_2_, the exponents α_1_, α_2_, χ_1_, and χ_2_, the universal behaviors of the kinetics, and the crossover between the two expansion stages are newly calculated in Supporting Information, as presented in Figures S14 to S18. The results agree with the theoretical predictions. For instance, τ_1_ exhibits a scaling behavior consistent with ϕ_0_^–8/3^. The exponent α_2_ possesses the distinctive 3D value of 1/5. These results demonstrate robustness of the theory, enabling explanation of the intricate kinetics of expansion in both 2D and 3D spaces.

It is worth noting that the theory proposed here can be extended to understanding the decompression of DNA molecules in a nanochannel. The dominant (second) stage of expansion is expected to exhibit a variation described by eq 3 with  in a one-dimensional (1D) space. Reccius et al.^14^ experimentally obtained a similar formula, expressed in our notation as . This equation depicts the expansion evolution from the initial chain size to the final size, by using the distinctive exponent 1/3 and a single time scale τ_d_. Jung and co-workers suggested two additional stages, an explosion stage followed by a subdiffusion stage, prior to the dominant global relaxation in the study of 1D expansion.^24^ The global relaxation was believed to follow exponential recovery. However, the relaxation curve obtained in their simulations appears to resemble the formula suggested by Reccius et al. in the long time regime. A careful investigation of the kinetic state diagrams, similar to Figure 4, thus should be conducted in the future to verify whether the dominant expansion in 1D space truly exhibits the power exponent 1/3 or not.

There are still many open questions that require further investigation. For example, the stiffness of the chain can have a great impact on expansion kinetics, especially when the persistence length becomes comparable to the confining dimension. It is known that some viruses have double-stranded DNA chains as their enclosed genomes. Therefore, it is necessary to study the expansion of semiflexible chains upon release in the next step. Hydrodynamics also play a crucial role and can significantly alter the expansion behaviors via, for example, accompanied inward flows. Another relevant area of research is studying how attractive interactions can slow down the expansion. It is anticipated that local attractive interactions within the chain can enhance the effect of entanglement, resulting in a significant moderation of the expansion in the first expansion stage.

## Supporting Information Available

Derivations of theory, simulation details, snapshots, additional simulation results, and reanalysis of the simulation data in 3D space (PDF)
