# Supplementary material for: Expansion Kinetics of Flexible Polymers upon Release from a Disk-Shaped Confinement
Source: ACS Omega. 2024 Mar 8;9(12):13797–802. doi: 10.1021/acsomega.3c08378 (PMC10975584; doi:10.1021/acsomega.3c08378)
Supplement: Supplementary file 1 — ao3c08378_si_001.pdf [file ao3c08378_si_001.pdf]

Supporting Information: Expansion Kinetics of Flexible Polymers upon Release from a Disk-Shaped Confinement

Pai-Yi Hsiao[1,2*]
[1]Department of Engineering and System Science,
National Tsing Hua University, Hsinchu, Taiwan, R.O.C.
[2]Institute of Nuclear Engineering and Science, National Tsing Hua University, Hsinchu, Taiwan, R.O.C.
*Corresponding author, email: pyhsiao@ess.nthu.edu.tw; pyhsiao@mx.nthu.edu.tw

S1. DERIVATION OF CHAIN-SIZE EVOLUTION IN THE TWO EXPANSION STAGES

The balance between the rate of free energy change and the rate of energy dissipation allows us to study the kinetics of expansion using the equation

$$\frac{\mathrm{d}R}{\mathrm{d}t} = -\frac{1}{\eta_{\mathrm{eff}}}\frac{\mathrm{d}F}{\mathrm{d}R} \tag{S1}$$

where R represents the chain size and η_{eff} is an effective friction coefficient associated with the expansion speed $\frac{\mathrm{d}R}{\mathrm{d}t}$. In the first stage of expansion, the free energy is estimated to be $F \sim k_{\mathrm{B}}T\left(\frac{\sigma N^{\nu_{\mathrm{b}}}}{R}\right)^{z}$ with $z = \frac{d}{d\nu_{\mathrm{b}}-1}$ in a d-dimensional space. Eq. (S1) thus gives

$$\frac{\mathrm{d}R}{\mathrm{d}t} \sim -\left(\frac{k_{\mathrm{B}}T}{\eta_{\mathrm{eff}}\sigma}\right)N^{z\nu_{\mathrm{b}}}\left(\frac{R}{\sigma}\right)^{-z-1} \times (-z) \tag{S2}$$

$$\sim \left(\frac{k_{\mathrm{B}}T}{\eta_{\mathrm{eff}}\sigma}\right)N^{z\nu_{\mathrm{b}}}\left(\frac{R}{\sigma}\right)^{-(z+1)} \tag{S3}$$

The factor z appearing at the end of Eq. (S2), due to the differentiation, can be disregarded when studying scaling, in order to avoid distraction. It can be considered as part of the prefactor in Eq. (S3), which is not explicitly expressed there. Given the initial chain size R_0, the differential equation can be solved:

$$\left(\frac{R}{\sigma}\right)^{z+2} - \left(\frac{R_0}{\sigma}\right)^{z+2} \sim \left(\frac{k_{\mathrm{B}}T}{\eta_{\mathrm{eff}}\sigma^2}\right)N^{z\nu_{\mathrm{b}}}t. \tag{S4}$$

Here, the factor $\frac{1}{z+1}$, which is related to the integration on the left-hand side of the equation, has been omitted following the same principle used in scaling derivation. We can rewrite the equation in a more precise form:

$$\left(\frac{R}{\sigma}\right)^{z+2} \simeq \left(\frac{R_0}{\sigma}\right)^{z+2} + c_1\left(\frac{k_{\mathrm{B}}T}{\eta_{\mathrm{eff}}\sigma^2}\right)N^{z\nu_{\mathrm{b}}}t \tag{S5}$$

by reintroducing the prefactor, referred to as c_1. The equation is then simplified to

$$R \simeq R_0\left[1 + \frac{t}{\tau_1}\right]^{\alpha_1} \tag{S6}$$

where $\alpha_1 = \frac{1}{z+2} = \frac{d\nu_{\mathrm{b}}-1}{2(d\nu_{\mathrm{b}}-1)+d}$ and $\tau_1 = c_1^{-1}\left(\frac{\eta_{\mathrm{eff}}\sigma^2}{k_{\mathrm{B}}T}\right)N^{-z\nu_{\mathrm{b}}}\left(\frac{R_0}{\sigma}\right)^{z+2}$. For a well-confined chain, the initial chain size R_0 scales with the cavity diameter D. Therefore, we can relate R_0 to the confining volume fraction ϕ_0 using the relation $\frac{R_0}{\sigma} \sim \frac{D}{\sigma} = \left(\frac{N}{\phi_0}\right)^{1/d}$. Assuming $\eta_{\mathrm{eff}} \sim \eta_0 N^{1+\chi_1}$, we obtain $\tau_1 \simeq A_1\left(\frac{\eta_0\sigma^2}{k_{\mathrm{B}}T}\right)N^{1+\chi_1-z\nu_{\mathrm{b}}+\frac{z+2}{d}}\phi_0^{-\frac{z+2}{d}}$, which can be deduced to be $\tau_1 \simeq A_1\left(\frac{\eta_0\sigma^2}{k_{\mathrm{B}}T}\right)N^{\frac{2}{d}+\chi_1}\phi_0^{-\left(\frac{2}{d}+\frac{1}{d\nu_{\mathrm{b}}-1}\right)}$. Since $\frac{\eta_0\sigma^2}{k_{\mathrm{B}}T}$ represents the Rouse time, which is responsible for the time unit, we know that A_1 is indeed a dimensionless prefactor.

In the second stage of expansion, the chain adopts a coil conformation and the Flory free energy $F \sim k_{\mathrm{B}}T\left(\frac{R^2}{N\sigma^2} + \frac{\sigma^d N^2}{R^d}\right)$ is utilized. The kinetics can be solves from the equation, $\frac{\mathrm{d}R}{\mathrm{d}t} + \frac{k_{\mathrm{B}}T}{\eta_{\mathrm{eff}}}\frac{2R}{N\sigma^2} - \frac{k_{\mathrm{B}}T}{\eta_{\mathrm{eff}}}\frac{d\sigma^d N^2}{R^{d+1}} \sim 0$, derived from Eq. (S1). It takes the basic form of a Bernoulli differential equation as $\frac{\mathrm{d}R}{\mathrm{d}t} + aR = bR^p$, and the solution is given by

$R = \left(\frac{b}{a}\right)^{\frac{1}{1-p}} \left[1 - c\, e^{-(1-p)at}\right]^{\frac{1}{1-p}}$ where c is a factor. Therefore, the evolution of the chain size can be solved and takes the form as a recovery function powered by a specific exponent $\alpha_2 = \frac{1}{d+2}$:

$$R \simeq R_\mathrm{F} \left[1 - a_c \exp\left(-\frac{t}{\tau_2}\right)\right]^{\alpha_2} . \tag{S7}$$

In the solution, R_F represents the final chain size and the well-known scaling relation $R_\mathrm{F} \sim \sigma N^\nu$ with $\nu = \frac{3}{d+2}$ is recovered. The characteristic time τ_2 scales as $\frac{\eta_\mathrm{eff}\sigma^2}{k_\mathrm{B}T}N$. Assuming $\eta_\mathrm{eff} \sim \eta_0 N^{1+\chi_2}$, we have $\tau_2 \simeq A_2 \left(\frac{\eta_0\sigma^2}{k_\mathrm{B}T}\right) N^{2+\chi_2}$ where A_2 is the prefactor.

Eq. (S7) also includes a factor a_c, for which I propose setting the value to 1. This choice is based on the observations from simulations in order to effectively describe expansion behavior for the case of long chain with a high confining volume fraction. Further explanation will be provided at the end of Section S4, near Figure S11.

S2. SIMULATION DETAILS AND SNAPSHOTS

In this study, a chain is modeled by using a bead-spring chain approach, and the 2D expansion behavior of the chain is investigated within a slit confinement. To comply with the geometrical constraint in a two-dimensional space, the slit height H has to be sufficiently small to prevent the occurrence of passing of one portion of the chain over the other portion in the confinement. The threshold height for the passing-over is $H_* = 1 + \frac{\sqrt{3}}{2}$, given a unit bead diameter. It is determined to ensure that a bead can pass through the upper cleavage space created by the connected beads near the bottom wall illustrated in Figure S1. In my simulations, a value of $H = 1.8$ is used. This value is

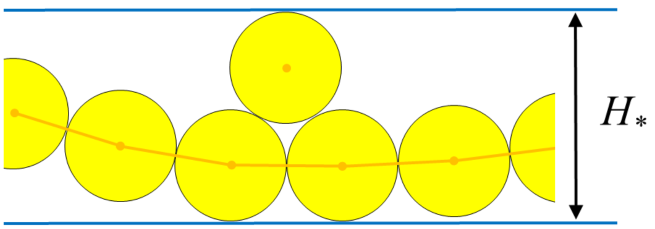

FIGURE S1: Illustration showing a critical situation where a monomer, the middle yellow bead near the top wall (represented as a blue line), is allowed to pass through the upper cleavage space created by the connected monomers near the bottom wall of a slit confinement. The threshold height of the slit can be calculated to be $H_* = 1 + \frac{\sqrt{3}}{2}$.

selected to be small enough to prevent the passing-over, while also allowing for the system to relax at a fast rate.

A disk-shaped cavity is created by enclosing it with a large cylindrical side wall perpendicular to the slit confinement. The diameter of the cylindrical wall is D. For each studied (N, ϕ_0) case, 1000 independent configurations of polymer confined in the disk cavity are prepared prior to the releasing process. A configuration is obtained by loading a chain through a small tube perpendicular to the confining disk, by pumping the monomers downward in the tube, as illustrated in Figure S2(a). After loading, the chain is equilibrated in the disk-shaped cavity (see Figure S2(b)) for a minimum of 10^8 timesteps. Random forces given by the Langevin thermostat are generated by using different seeding numbers in order to produce independent configurations.

Snapshots of chain expansion during a releasing process from $\phi_0 = 0.6$ are presented in Figure S3 . The top-view images show that the chain initially expands with a disk-like structure. It gradually evolves and adopts a coil conformation over time.

The bonding between adjacent monomers on a chain in the simulations is modeled using a harmonic potential with a large spring constant, k=6000 $[k_\mathrm{B}T/\sigma^2]$. The bond length, denoted by b, has been studied within the confining cavity during the equilibrating phase. The mean value is obtained: $\langle b \rangle = 1.00$ with a standard deviation of 0.01. The maximum b detected in all of the runs is 1.049956. For the selected slit height $H = 1.8$, the passing-over of a monomer through the upper cleavage space of two connected monomers at the bottom (refer to the schematic in Figure S1) would require a stretching of the bond length between the two monomers to be 1.2. This represents an extremely rare event and is highly unlikely to happen because it involves stretching the bond length by about twenty times the standard deviation. I note that there have been no detection of such passing-over events in any of the runs.

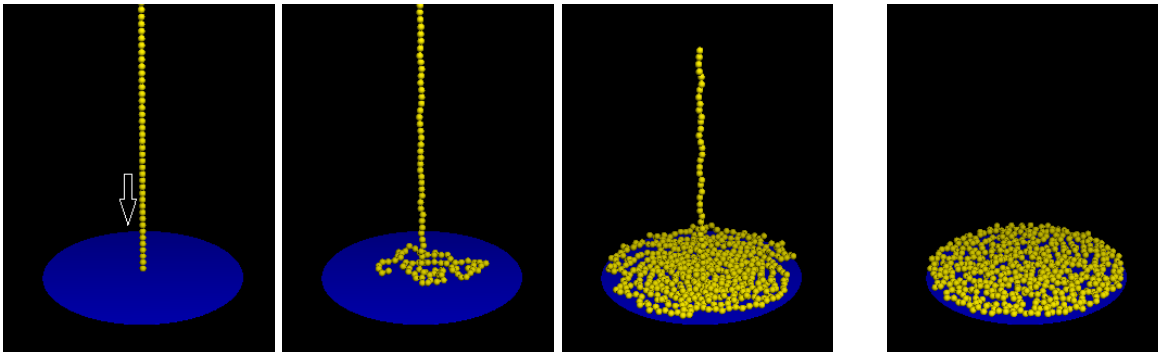

(a) loading (b) equilibrating

FIGURE S2: **(a)** Snapshots showing how a chain is loaded into a disk-shaped cavity. The chain is pumped downward through a small tube (not shown) perpendicular to the disk. The yellow beads represent the monomers and the blue circle indicates the bottom wall of the cavity. The top disk wall and the cylindrical side wall are not shown for clear visualization of the chain. **(b)** Snapshot of a chain equilibrated inside the disk-shaped cavity. The chain length is $N = 512$ and the confinement has $\phi_0 = 0.6$.

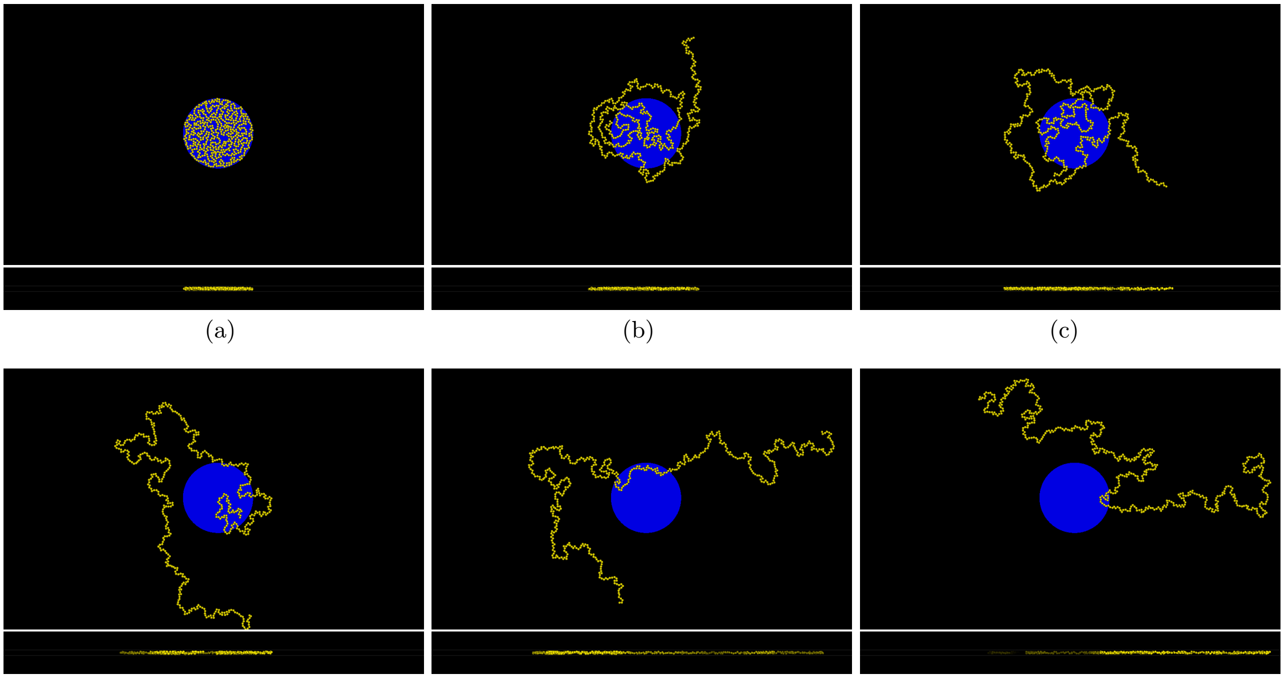

(a) (b) (c) (d) (e) (f)

FIGURE S3: Snapshots of a chain in the expanding phase at (a) $t = 0.0$, (b) $t = 4.5 \times 10^4$, (c) $t = 2.3 \times 10^5$, (d) $t = 6.3 \times 10^5$, (e) $t = 2.3 \times 10^6$, and (f) $t = 6.5 \times 10^6$. The upper wide pictures in the panels present the top-view images of the system, while the lower narrow ones show the side-view images. The monomers are represented by yellow beads. The blue circle in a upper picture indicates the location of the cavity which confines the chain prior to the release. The chain length is $N = 512$ and the confining condition is $\phi_0 = 0.6$.

For reference, the probability density distribution of bond length, $p(b)$, is plotted in Figure S4. Panel (a) displays the distribution for $\phi_0 = 0.6$ while varying the chain length N. Panel (b) shows $p(b)$ for $N = 512$ by varying ϕ_0. We can see that the distribution curves appear nearly identical for different values of N and ϕ_0.

I remark that despite the utilization of the large spring constant, the chain retains its flexibility due to the modeling of the monomer beads using the Weeks-Chandler-Anderson potential, which does not involve any frictional force between the beads. As a result, a bead is able to move freely in any direction tangent to the connected bead while

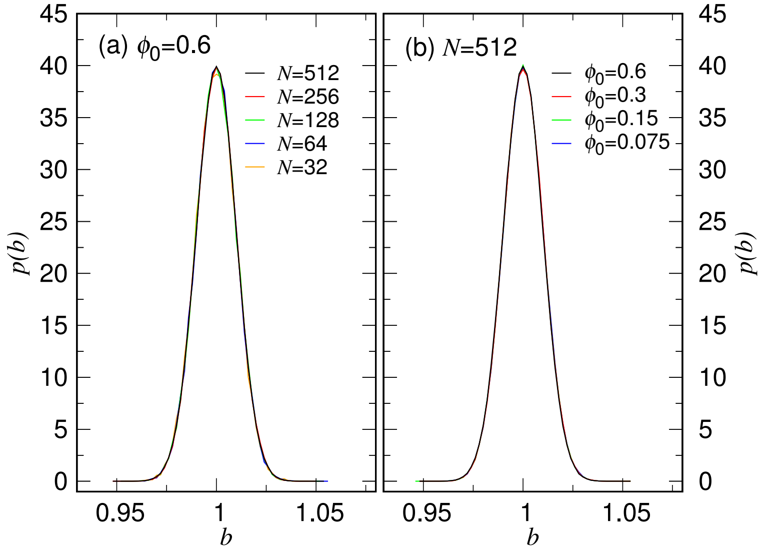

FIGURE S4: **(a)** Probability density distribution $p(b)$ of bond length plotted for $\phi_0 = 0.6$ while varying the chain length N. **(b)** Distribution $p(b)$ for $N = 512$ plotted by varying ϕ_0.

maintaining the bond length of about 1.0. Therefore, the chain remains flexible.

S3. EVOLUTION OF CHAIN SIZE PLOTTED ON LINEAR SCALE

The chain size is characterized by calculating the radius of gyration, defined by $R_{\rm g} = \left(\frac{1}{N}\sum_{i=1}^{N}(\mathbf{r}_i - \mathbf{r}_{\rm cm})^2\right)^{1/2}$. Figure S5(a) illustrates the time evolution of $R_{\rm g}$ for $N = 512$ in five single runs, released from the initial confinement condition $\phi_0 = 0.6$. These curves are plotted using colored thin lines. Fluctuations of the curves can be smoothed out by calculating the average $R(t) = \langle R_{\rm g}^2\rangle^{1/2}$ over 1000 single runs. The average curve has been plotted by using the thick black line in the figure for comparison.

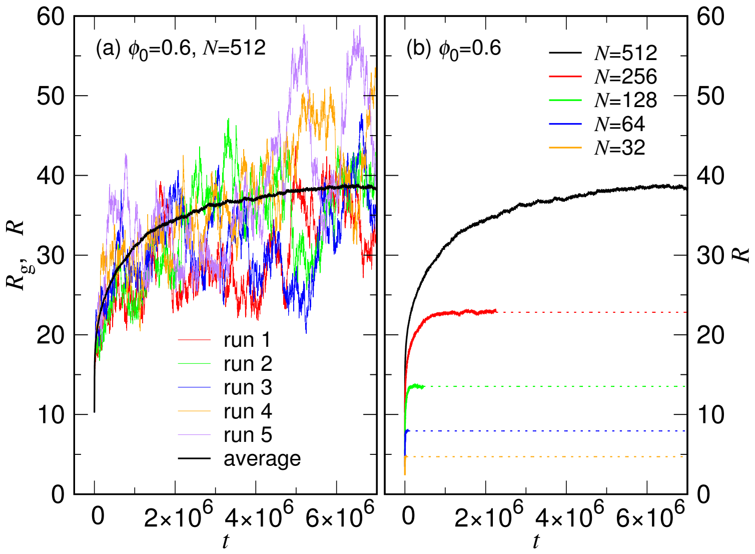

FIGURE S5: **(a)** Evolution of chain size, $R_{\rm g}$, in five single runs represented by colored thin lines. For comparison, the average curve over 1000 independent single runs is plotted using the black thick line. **(b)** The average curves for different chain lengths released from the confining condition of $\phi_0 = 0.6$.

The variations of the average curves with respect to different chain lengths are presented in Figure S5(b). Initially, the chain exhibits rapid expansion, leading to a sharp increase in the evolution curve that may not be clearly discernible on a linear scale. On the other hand, the recovery of the chain to its natural size during the later stage requires a relatively longer time, which is strongly dependent on the chain length N. Therefore, studying the expansion behavior on a logarithmic scale, as shown in Figure 1(a) in the paper, is more suitable.

The average curves for the case of $(N, \phi_0) = (512, 0.6)$ calculated from 10, 100, and 1000 independent single runs are presented in Figure S6, plotted using both a linear and a logarithmic time scale. One can observe that the fluctuations decrease as the number of runs involved in the averaging increases. Notably, significant fluctuations persist still in the average curve over 100 runs. It indicates that tens of measurements of individual expansion curves are usually insufficient for the study of the kinetics with a high level of precision.

In this study, the damping time parameter for the Langevin thermostat is set as $\tau_{\rm L} = 0.05$. Recall that the physical quantities are described in terms of the simulation units where m represents mass, $k_{\rm B}T$ represents energy, and σ represents length. To map the simulation to a real system, like a single-strand DNA chain, one can choose the following values: $m = 320\text{g/mol} = 5.33 \times 10^{-25}\text{kg}$, $k_{\rm B}T = 300k_{\rm B} = 4.14 \times 10^{-21}\text{J}$, and $\sigma = 3.4\text{Å} = 3.4 \times 10^{-10}\text{m}$. Using these values, the simulation time unit is $t_{\rm u} = \sigma\sqrt{\frac{m}{k_{\rm B}T}} = 3.86\text{ps}$. The dynamic viscosity ξ of solvent can be then estimated from the Stokes' law $\eta_0 = 3\pi\sigma\xi$, where the friction coefficient η_0 of a monomer is equal to $\frac{m}{\tau_{\rm L}} = 2.76 \times 10^{-12}\text{kg}\cdot\text{s}^{-1}$. The obtained value is $\xi = 0.862\text{cP}$, which aligns with the dynamic viscosity of water at room temperature. Therefore, this model effectively simulates expansion of a ssDNA chain in an aqueous environment. It is expected to exhibit comparable variations and fluctuations in behavior.

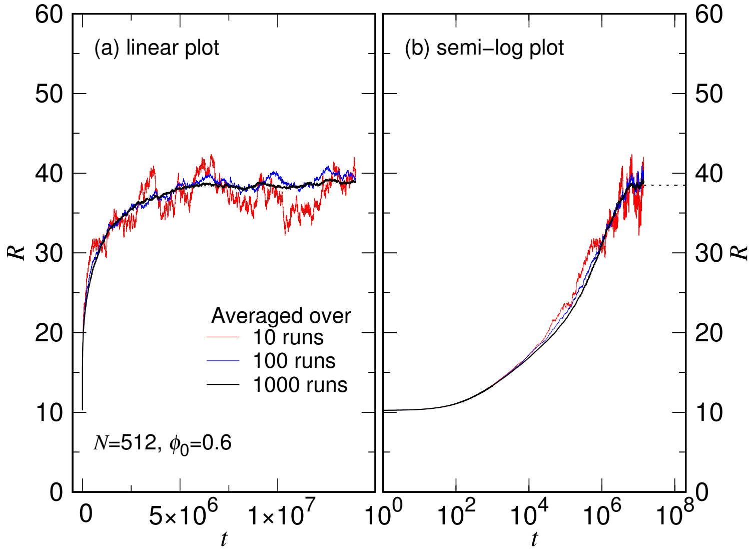

FIGURE S6: Time evolution curves averaged from 10, 100, and 1000 independent runs for $N = 512$ released from $\phi_0 = 0.6$, plotted using (a) a linear and (b) a logarithmic time scale.

S4. COLLAPSE OF $\hat{R}$- AND $\tilde{R}$-CURVES AND SEARCH FOR ω_1 AND ω_2 FOR THE BEST COLLAPSE

The scaling behavior of the R curves are studied by calculating the two quantities: (1) $\hat{R} \equiv \frac{R}{R_0}$ and (2) $\tilde{R} \equiv \frac{R}{R_F}$. The first quantity measures the expansion ratio and evolves from the initial value of 1. The second one describes the progress for the completion of the expansion and tends toward the final value, one. The two kinds of curves can be horizontally shifted by multiplying time by a factor $\omega_{1,2}^{9-g}$. Here ω_1 and ω_2 are two parameters that determine the amount of shift applied to the respective curves, and the value of $g = \log_2 N$ represents the exponent associated with the change of chain length in the study. By appropriately selecting values for ω_1 and ω_2, the curves in the small and in the large time regions can be separately collapsed onto the one for $N = 512$.

The values were determined by calculating the mean distribution widths, $\langle W_1 \rangle$ and $\langle W_2 \rangle$, for the two sets of collapsed curves in the respective time regions, $t_{\omega_1} < 10\tau_1$ and $t_{\omega_2} > 0.5\tau_2$. The minimum mean widths were then searched. Figure S7 illustrates how the widths of the collapse in the two regions change with the choice of ω_1 and ω_2 for the case $\phi_0 = 0.6$. In the middle panel of Figure S7(a), we can see that $\omega_1 = 2.03$ gives a small width of

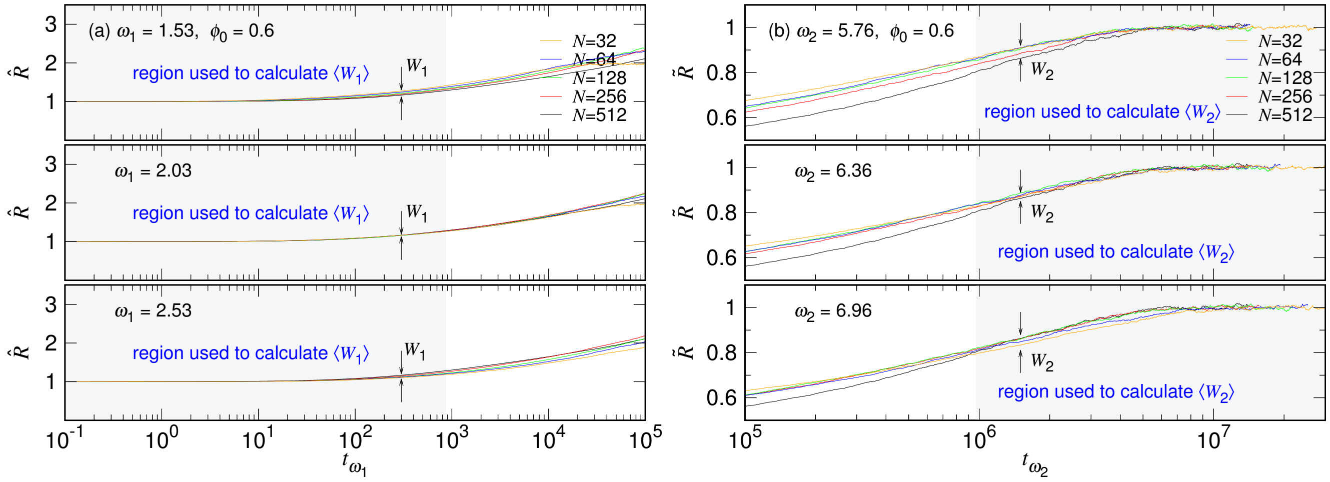

FIGURE S7: **(a)** Collapse of the $\hat{R}$ curves plotted against $t_{\omega_1} = t \times \omega_1^{9-g}$ using three different ω_1 values, 1.53, 2.03, and 2.53. The mean collapse width $\langle W_1 \rangle$ is calculated within the light-gray region, $t_{\omega_1} < 10\tau_1$. **(b)** Collapse of the $\tilde{R}$ curves plotted against $t_{\omega_2} = t \times \omega_2^{9-g}$ using three different ω_2 values, 5.76, 6.36, and 6.96. The mean collapse width $\langle W_2 \rangle$ is calculated within the light-gray region, $t_{\omega_2} > 0.5\tau_2$. Here $g = \log_2 N$ is the exponent associated with the change of chain length in the study.

collapse within the time region $t_{\omega_1} < 10\tau_1$ (in light-gray color), compared to $\omega_1 = 1.53$ in the top panel, which yields a larger width owing to an insufficient shift of the $\hat{R}$ curves along the time axis, and $\omega_1 = 2.53$ in the bottom panel, which gives too much shift. For the second expansion stage, a small mean width $\langle W_2 \rangle$ of collapse can be achieved by choosing $\omega_2 = 6.36$, as shown in the middle panel of Figure S7(b) within the light-gray region. Decreasing or increasing the value of ω_2 results in under-shift or over-shift of the curves, respectively, leading to a larger distribution width of those $\tilde{R}$ curves, as seen in the top and the bottom panels.

The calculated mean widths $\langle W_1 \rangle$ vs. ω_1 and $\langle W_2 \rangle$ vs. ω_2 are plotted in Figure S8 for various ϕ_0. The ω_1 and ω_2 values for the occurrence of the minimum mean width, accurate to the second decimal place, have been reported in Figure 2 of the paper, and in the next two figures, Figure S9 and S10.

Figure S9 is a replot of Figure 2 in the paper, with an enlarged time region for small $\hat{R}$ and an enlarged time region for large $\tilde{R}$. The zoom-in views provide a clearer visualization of the collapse of the curves and the corresponding fit for the two releasing cases with $\phi_0 = 0.6$ and $\phi_0 = 0.3$.

The collapses of the two sets of curves for the cases of loose confinement, $\phi_0 = 0.15$ and 0.075, are shown in Figure S10. As we can see, the procedures still work well, even if ϕ_0 is small. The fitting parameters α_1, τ_1, α_2, and τ_2 are reported in the respective panels in the figure.

In the simulations, it is observed that the set of the chain size evolution curves for a give length N, but released from different confining conditions ϕ_0, collapse together as t increases, specifically when t becomes comparable to the second characteristic time τ_2 (refer to Figure S11(a)). Moreover, the $R(t)$ curves progressively curve downwards in the small time region as ϕ_0 increases and approach a limiting curve. This limiting curve can be interpreted as deducing from the extreme scenario of $\phi_0 \to \infty$. In this hypothetical situation, the chain should have a size of zero

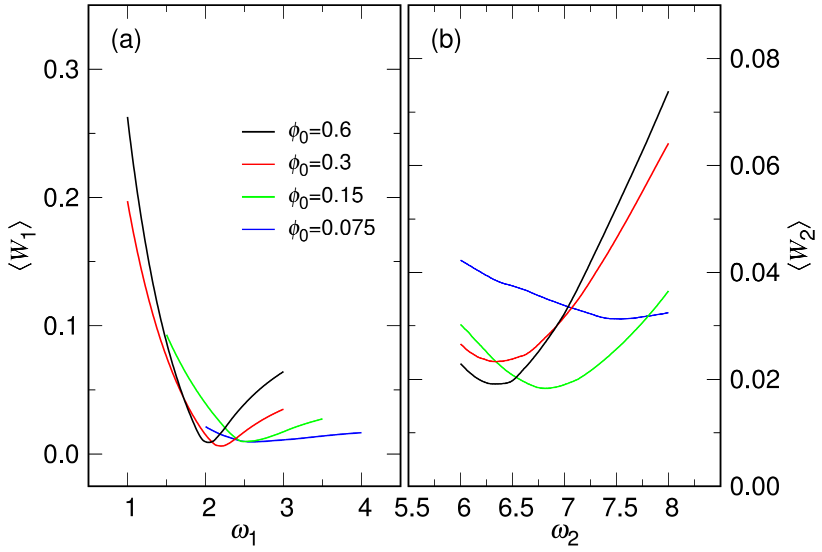

FIGURE S8: **(a)** Mean distribution width $\langle W_1 \rangle$ of the collapse curves in the first expansion region as a function of the parameter ω_1. **(b)** Mean distribution width $\langle W_2 \rangle$ of the collapse curves in the second expansion region as a function of the parameter ω_2.

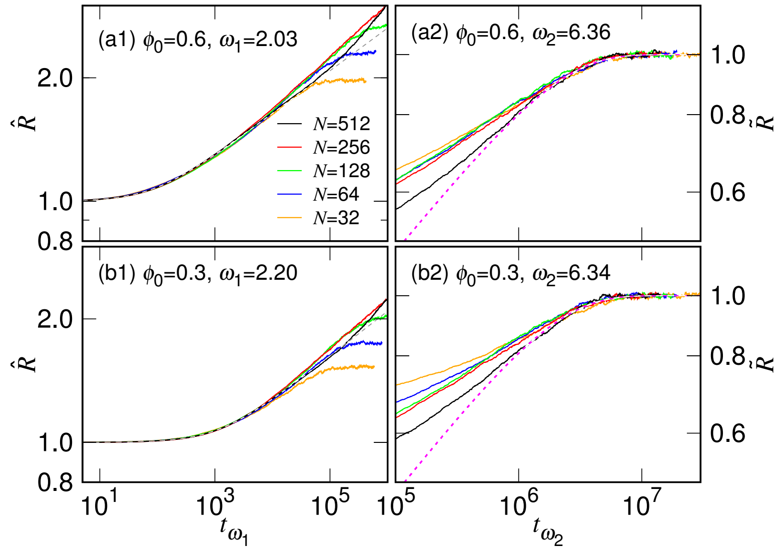

FIGURE S9: Replot of Figure 2 in the paper, with enlargements in the relevant regions to provide a clearer visualization of the curve collapses and fits. Panels (a1,b1) display $\hat{R}$ versus t_{ω_1} and the corresponding fitting for $\phi_0 = 0.6$ and 0.3, respectively. Panels (a2,b2) present $\tilde{R}$ versus t_{ω_2} and the fitting for the same cases.

at the starting point, $t = 0$, which requires setting $a_c = 1$ in Eq. (S7), or Eq. (3) in the paper, to define the limiting curve. To illustrate it, the function $\tilde{R}(t) = \left[1 - \exp\left(-\frac{t}{\tau_2}\right)\right]^{\alpha_2}$ is plotted for $N = 512$ in Figure S11(b) as the magenta dash-dotted line. The figure presents the normalized chain size $\tilde{R}$ versus t on a semilog scale. We can see that the function represents an appropriate limiting curve for the simulation curves, effectively describing the variations of chain size in the large time region, *i.e.* in the second expansion stage. It is important to note that the setting should be applicable for the case of long chains with a high confining volume fraction. However, in the simulations, not all values of N and ϕ_0 correspond to the case. The constant use of $a_c = 1$ allows us to test the robustness of the analysis. Despite this approximation, the results remain consistent and satisfactory, demonstrating the validity of using $a_c = 1$ in the study.

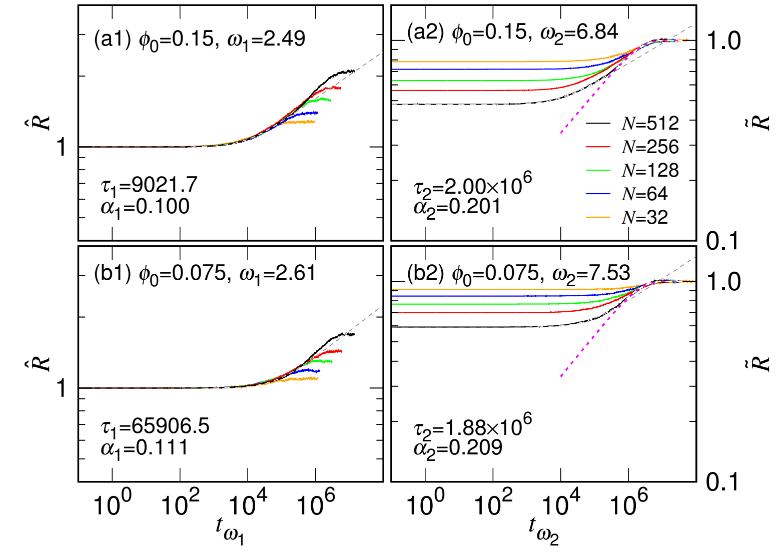

FIGURE S10: $\hat{R}$ vs. t_{ω_1} and $\tilde{R}$ vs. t_{ω_2} for (a1,a2) $\phi_0 = 0.15$ and (b1,b2) $\phi_0 = 0.075$. Recall the definitions: $\hat{R} = \frac{R}{R_0}$, $\tilde{R} = \frac{R}{R_\mathrm{F}}$, $t_{\omega_1} = t \times \omega_1^{9-g}$, and $t_{\omega_2} = t \times \omega_2^{9-g}$. The chain length N can be found in the legend of Panel (a2). The optimal values of ω_1 and ω_2 for achieving the best collapse, as well as the fit parameters α_1, τ_1, α_2, and τ_2, are reported in the respective panels. The fitting curves for the first and second expansion stages are represented by gray and magenta dashed lines, respectively.

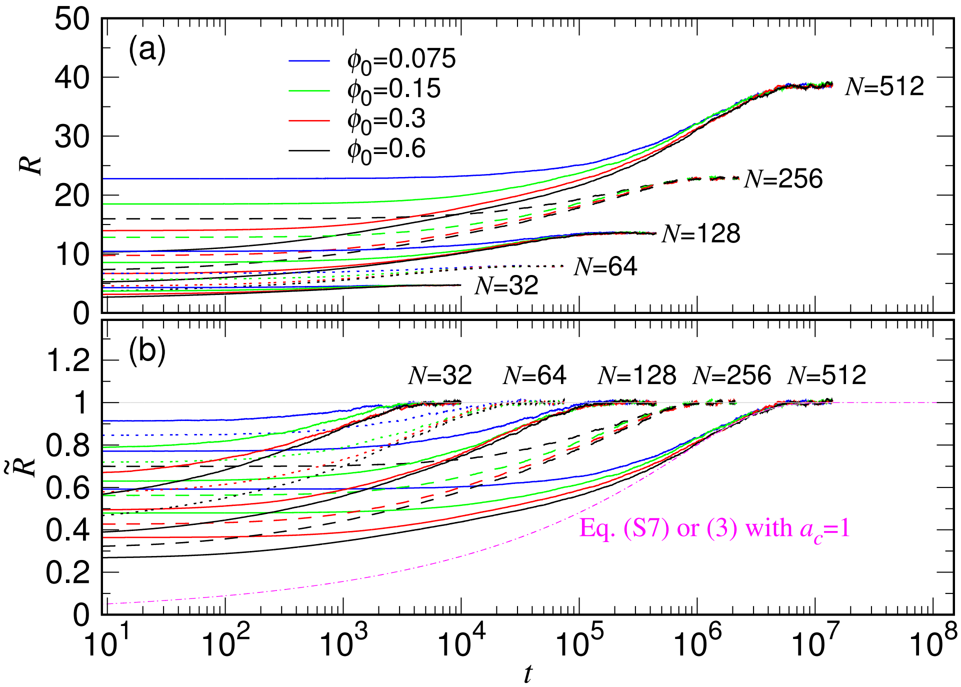

FIGURE S11: **(a)** Evolution curves $R(t)$ of chain size are plotted at a given N for various ϕ_0, as indicated in the legend of the panel. The value of N is provided near the set of the curves corresponding to this chain length. **(b)** The same plot as in panel (a), but with the chain size replaced by the normalized chain size $\tilde{R} = R/R_\mathrm{F}$. The magenta dash-dotted line represents the predicted curve for the case of $N = 512$, by using Eq. (S7) (or Eq. (3) in the paper) with $a_c = 1$.

S5. KINETICS OF EXPANSION

The kinetics of expansion can be studied by calculating the speed of expansion $V = \mathrm{d}R/\mathrm{d}t$ as a function of the transition coordinate, the chain size R. According to my theory, the speed should behave as

$$V \simeq \alpha_1 \frac{R_0}{\tau_1} \left(\frac{R}{R_0}\right)^{1-\frac{1}{\alpha_1}} \tag{S8}$$

in the first stage. Figure S12(a) plots $\mathrm{d}\hat{R}/\mathrm{d}t$ vs. $\hat{R}$ for $\phi_0 = 0.6$. We can see that the curves show scaling variations in the small $\hat{R}$ region and are parallel to each other for different N with a scaling exponent equal to $-8.68(7)$.

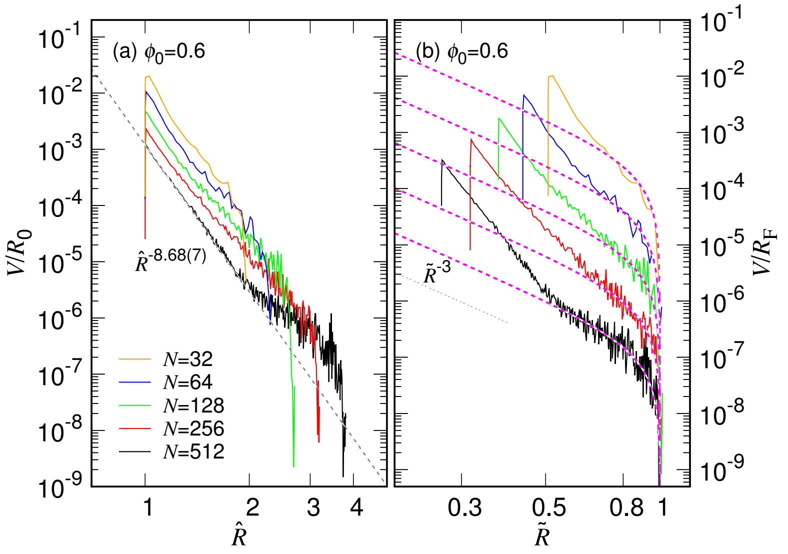

FIGURE S12: **(a)** $\mathrm{d}\hat{R}/\mathrm{d}t$ (or equivalently V/R_0) vs. $\hat{R}$. **(b)** $\mathrm{d}\tilde{R}/\mathrm{d}t$ (or equiv. V/R_F) vs. $\tilde{R}$. The confining condition before release is $\phi_0 = 0.6$. The chain length N can be read from the legend in Panel (a). The gray dashed line shows the power-law behavior in the 1st expansion stage while the magenta dashed lines indicate the predicted behaviors in the 2nd stage.

Similarly, in the second stage of expansion, the theory predicts

$$V \simeq \alpha_2 \frac{R_\mathrm{F}}{\tau_2} \left[\left(\frac{R}{R_F}\right)^{1-\frac{1}{\alpha_2}} - \left(\frac{R}{R_F}\right) \right]. \tag{S9}$$

Figure S12(b) presents the calculation results for $\frac{\mathrm{d}\tilde{R}}{\mathrm{d}t}$ versus $\tilde{R}$. The theoretical curves, $\frac{\mathrm{d}\tilde{R}}{\mathrm{d}t} = \frac{\alpha_2}{\tau_2}\left[\tilde{R}^{1-\frac{1}{\alpha_2}} - \tilde{R}\right]$, for different values of N have been plotted as magenta dashed lines for comparison, with α_2 set to the 2D value of 0.25. These curves exhibit an asymptotic behavior of $\tilde{R}^{-3}$ as $\tilde{R}$ becomes small. Due to the smaller value of α_1 compared to α_2, the speed profile in the log-log plot exhibits a change in slope from $1 - \frac{1}{\alpha_1}$ to $1 - \frac{1}{\alpha_2}$. This transition is clearly observed in the case of $N = 512$ near $\tilde{R} = 0.5$. As a result, the second-stage expansion displays a distinctive hip curve, rapidly decreasing to zero as $\tilde{R}$ approaches one.

The crossover between the first and second stages of expansion can be studied by equating the expansion speed given by Eq. (S8) and Eq. (S9), which defines the chain size R_* at crossover. The R_* is then used to determine the corresponding crossover time t_* on the plot of R versus t, as shown in Figure S13, where the open circle symbols denotes the crossover points found. We can see that these crossover points align basically on a straight line on the log-log plot, exhibiting a power-law behavior as $R_* \sim t_*^{0.326(19)}$. On the left of the line is the region for the occurrence of the first expansion stage, while on the right is the region for the one of the second stage.

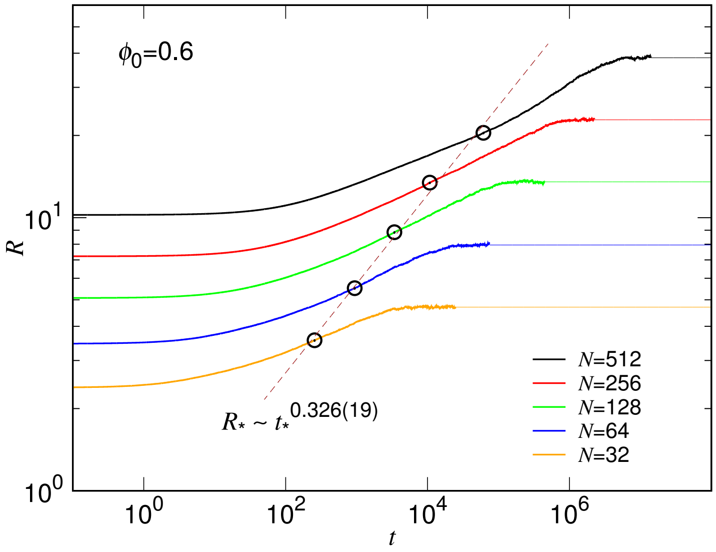

FIGURE S13: Expansion chain size R vs. t for the case of $\phi_0 = 0.6$. The open circle symbols indicate the crossover points between the first and the second expansion stages for a given chain lengths N. These points exhibit a scaling relation $R_* \sim t_*^{0.326(19)}$.

S6. REANALYZING THE SIMULATION DATA IN 3D SPACE

The methods and procedures described in this study are utilized to reanalyze the expansion data of polymers released from a spherical cavity in a three-dimensional space (original data published in Polymers 15 (2023) 198). The reanalysis is conducted to verify the applicability of the theory and the methods across different spatial dimensions.

The new calculations for the collapses of the size evolution curves in the first and in the second expansion stages for the 3D simulations are given in Figure S14. Four confining volume fractions, $\phi_0 = 0.4$, 0.2, 0.1, and 0.05, in the three

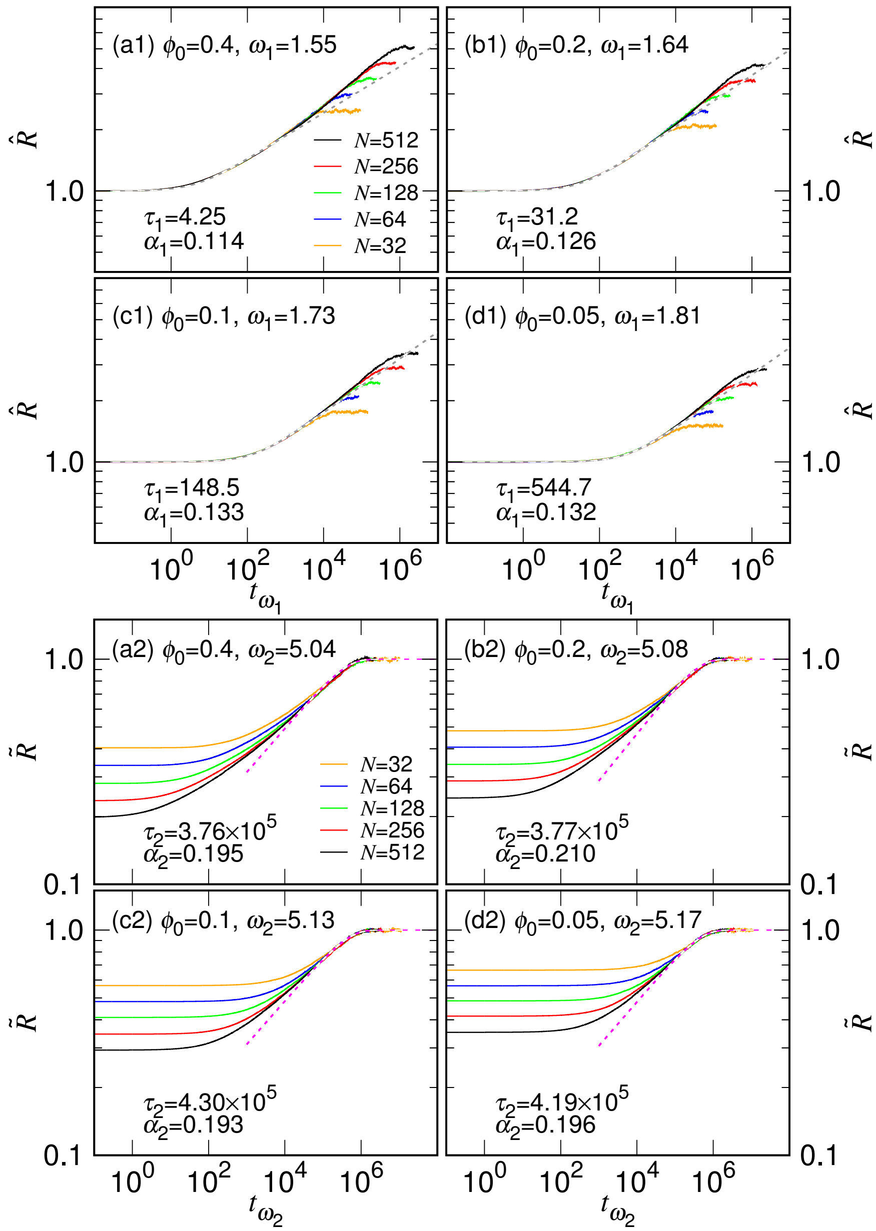

FIGURE S14: [3D results] $\hat{R}$ vs. t_{ω_1} and $\tilde{R}$ vs. t_{ω_2} for $\phi_0 = 0.4$, 0.2, 0.1, and 0.05 in the there-dimensional space. The definition of quantities are identical to the 2D cases: $\hat{R} = \frac{R}{R_0}$, $\tilde{R} = \frac{R}{R_F}$, $t_{\omega_1} = t \times \omega_1^{9-g}$, and $t_{\omega_2} = t \times \omega_2^{9-g}$. The chain length N can be found in the legend of Panel (a1) and (a2). The optimal values of ω_1 and ω_2 for achieving the best collapse, as well as the fit parameters α_1, τ_1, α_2, and τ_2, are reported in the respective panels. The fitting curves for the first and second expansion stages are represented by gray and magenta dashed lines, respectively.

dimensional space are investigated. The fits for the collapse of the first-stage curves are plotted as gray dashed lines atop of the data, while the ones for the second-stage curves are plotted as magenta dashed lines. The fit parameters

are reported in the corresponding panels in the figure.

Figure S15 presents the summarized study, concerning the variations of the characteristic times in the two expansion stages, τ_1 and τ_2, and the associated exponents, α_1 and α_2, with respect to the confining volume fraction ϕ_0. As

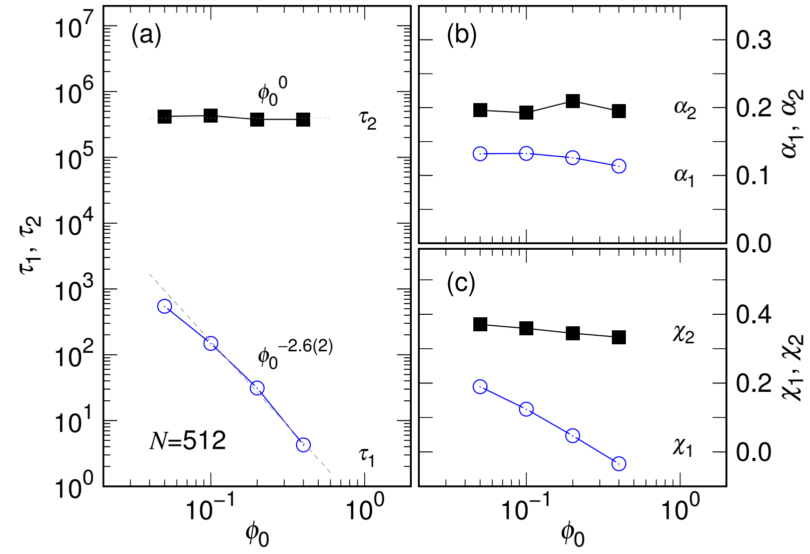

FIGURE S15: [3D results] (a) τ_1 and τ_2 vs. ϕ_0 for $N = 512$. **(b)** α_1 and α_2 vs. ϕ_0. **(c)** χ_1 and χ_2 vs. ϕ_0. The simulations were run in the three-dimensional space.

we can see in Panel (a), the characteristic time τ_1 scales approximately as $\phi_0^{-2.6(2)}$, which agrees with the predicted scaling $\phi_0^{-\frac{8}{3}}$ for $\nu_\mathrm{b} = 0.5$ given in Table 1 in the paper. The second characteristic time τ_2 is of several orders of magnitude larger than τ_1 and appears to be insensitive to the volume fraction ϕ_0 of the confinement, as predicted by the theory. Panel (b) shows that α_2 is roughly constant against ϕ_0, adopting about the predicted 3D value of 0.2, which is distinguishable from the 2D value, 0.25. On the other hand, the exponent α_1 displays a slight increasing trend from 0.11 to 0.13 as ϕ_0 decreases. The exponents χ_1 and χ_2 account for the additional dependence on N in the characteristic time beyond the predictions, resulting from the utilization of the single global speed to estimate the energy dissipation in Eq. (1) of the paper. They can be obtained by calculating $\chi_1 = \log_2(\omega_1) - \frac{2}{3}$ and $\chi_2 = \log_2(\omega_2) - 2$. The value of χ_1 increases as ϕ_0 decreases, showing a more significant change compared to χ_2 (see Figure S15(c)).

The kinetic equations are newly investigated from the 3D simulations for the case with $\phi_0 = 0.4$. The variations of the dimensionless speeds, $\frac{\mathrm{d}\hat{R}}{\mathrm{d}\hat{t}}$ vs. $\hat{R}$ and $\frac{\mathrm{d}\tilde{R}}{\mathrm{d}\tilde{t}}$ vs. $\tilde{R}$, are presented in Panel (a1) and (a2) of Figure S16. We can see that the three-dimensional study recovers the two featured universal behaviors, represented by the gray dashed and magenta dashed lines, respectively. On the logarithmic scales, the curves exhibit a slope of $-8.51(8)$ in the first stage. They then transition to show an asymptotic slope of about -4 when the expansion process enters the second stage (refer to Panel (a2)). Towards the end of the process, the speed decreases sharply to zero. Figures S16(b1) and S16(b2) present the variation curves of $\frac{\mathrm{d}\hat{R}}{\mathrm{d}t}$ vs. $\hat{R}$ and $\frac{\mathrm{d}\tilde{R}}{\mathrm{d}t}$ vs. $\tilde{R}$, respectively. The two plots provide a clear visualization of the individual kinetics in the first and second expansion stages.

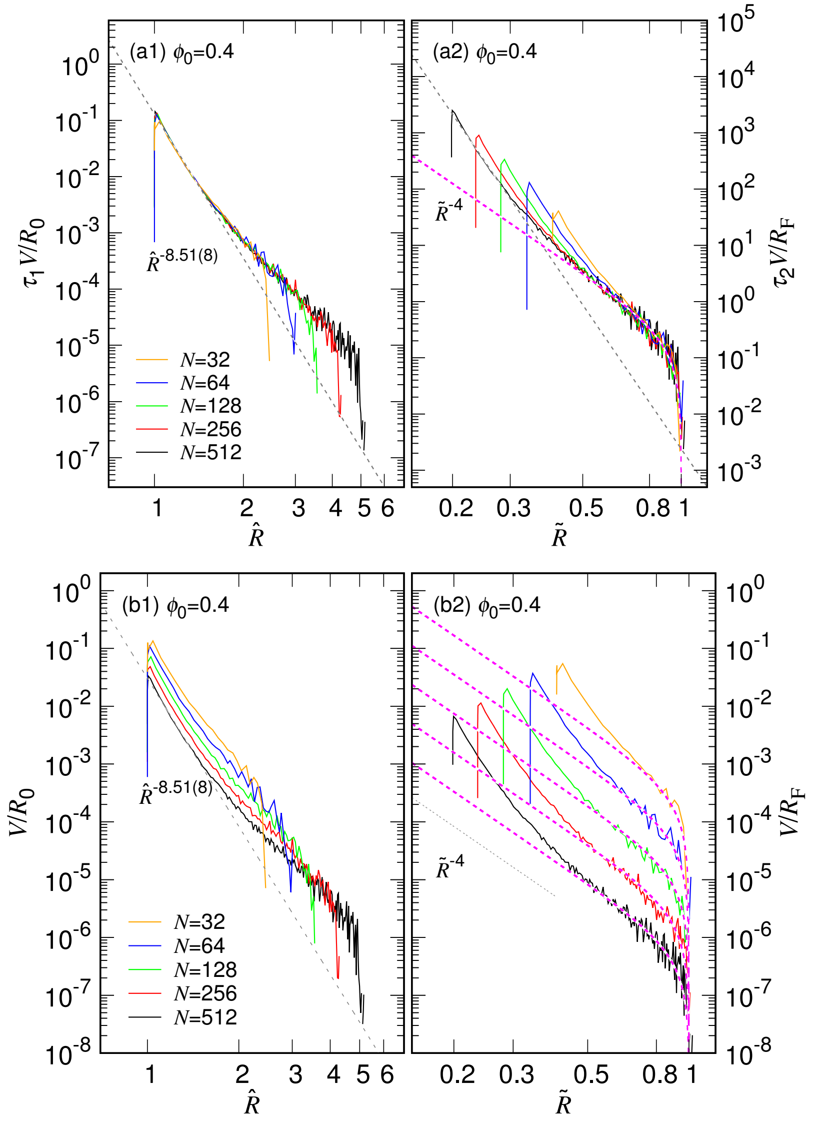

FIGURE S16: **[3D results]** **(a1)** $\mathrm{d}\hat{R}/\mathrm{d}\hat{t}$ (or equivalently $\tau_1 V/R_0$) vs. $\hat{R}$. **(a2)** $\mathrm{d}\tilde{R}/\mathrm{d}\tilde{t}$ (or equiv. $\tau_2 V/R_\mathrm{F}$) vs. $\tilde{R}$. **(b1)** $\mathrm{d}\hat{R}/\mathrm{d}t$ (or equiv. V/R_0) vs. $\hat{R}$. **(b2)** $\mathrm{d}\tilde{R}/\mathrm{d}t$ (or equiv. V/R_F) vs. $\tilde{R}$. The confining condition before release is $\phi_0 = 0.4$. Recall the definitions: $\hat{R} = \frac{R}{R_0}$, $\tilde{R} = \frac{R}{R_\mathrm{F}}$, $\hat{t} = \frac{t}{\tau_1}$, and $\tilde{t} = \frac{t}{\tau_2}$. The chain length N can be read from the legend in Panels (a1) and (b1). The gray dashed line shows the power-law behavior in the first expansion stage, while the magenta dashed lines indicate the predicted behaviors in the second stage. The simulations were run in the three-dimensional space.

The crossover size $\tilde{R}_*$ is determined by studying the intersection of the expansion speeds in the two stages, as shown by the brown and magenta dashed lines in Figure S17(a), in a way similar to treating the 2D cases. The obtained

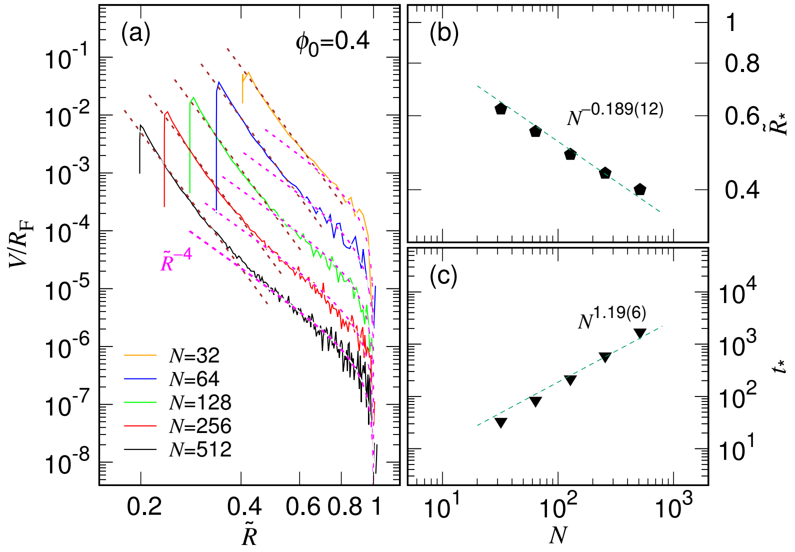

FIGURE S17: **[3D results]** **(a)** $d\tilde{R}/dt$ (or equiv. V/R_F) vs. $\tilde{R}$. The intersect between the two expansion speeds, represented respectively by the brown dashed line (Eq. (S8)) and the magenta dashed line (Eq. (S9), is used to determined the crossover size $\tilde{R}_*$ for each N. **(b)** The obtained $\tilde{R}_*$ is plotted against N. **(c)** t_* vs. N where the crossover time t_* is determined by mapping the associated size R_* on the $R(t)$ curves in Figure S18. The confining condition is $\phi_0 = 0.4$. The simulations were run in the three-dimensional space.

$\tilde{R}_*$ exhibits a scaling relation with the chain length, as $\tilde{R}_* \sim N^{-0.189(12)}$ (see Figure S17(b)). The crossover time t_* can then be determined with the help of the $R(t)$ curves obtained from the simulations in Figure S18 by mapping the value $R_* = R_F \tilde{R}_*$. Figure S17(c) shows that the resulting t_* also follows a power law, as $N^{1.19(6)}$. As a consequence,

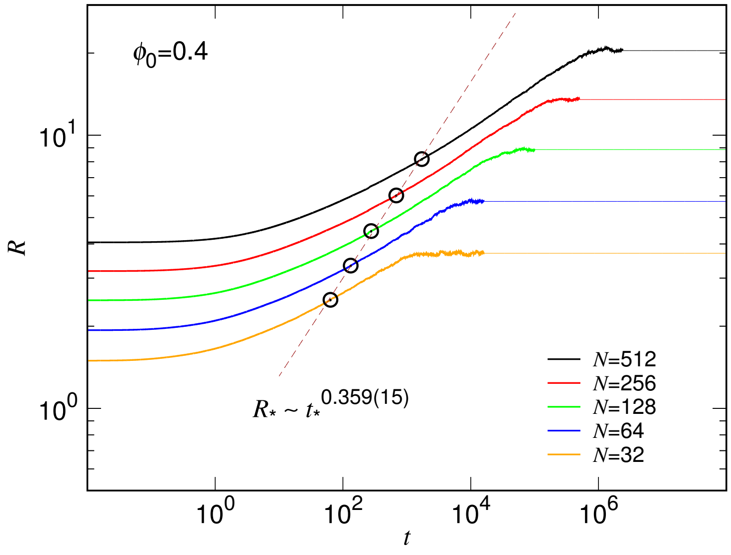

FIGURE S18: **[3D results]** Expansion chain size R vs. t for $\phi_0 = 0.4$. The open circle symbols indicate the crossover points between the first and the second expansion stages for a given chain lengths N. These points exhibit a scaling relation $R_* \sim t_*^{0.359(15)}$.

the crossover points in Figure S18, indicated by an open circle symbol, appear to fall on a straight line on the log-log

scale. It defines a demarcation line which takes the form of $R_* \sim t_*^{0.359(15)}$. On the left of the line depicts the region for the first expansion stage, whereas on the right depicts the one for the second stage.
